# Liberties, rights, public policies and water fluoridation

**DOI:** 10.11606/s1518-8787.2020054001804

**Published:** 2020-05-22

**Authors:** Antonio Carlos de Souza, Paulo Frazão

**Affiliations:** I Universidade de São Paulo Faculdade de Saúde Pública Programa de Pós-Graduação em Saúde Pública São PauloSP Brasil Universidade de São Paulo. Faculdade de Saúde Pública. Programa de Pós-Graduação em Saúde Pública. São Paulo, SP, Brasil; II Universidade de São Paulo Faculdade de Saúde Pública Departamento de Política, Gestão e Saúde São PauloSP Brasil Universidade de São Paulo. Faculdade de Saúde Pública. Departamento de Política, Gestão e Saúde. São Paulo, SP, Brasil

**Keywords:** Public Health Policies, Administrative Right, Sanitation Right, Socioeconomic Rights, Liberty

## Abstract

**OBJECTIVE:**

To discuss the negative and positive concepts of liberty and postulate its interdependent and complementary relationship in the evaluation of public policy intersectoral actions, taking water fluoridation as a case.

**METHOD:**

To describe scopes and limits regarding 1950s Isaiah Berlin’s distinction, showing its validity in facing the harmful effects of an uncontrolled market economy and an autocratic political regime.

**RESULTS:**

Both the rights that protect citizens against a powerful state and the rights that protect the state against powerful citizens were equally acknowledged as crucial.

**CONCLUSION:**

We argued that, in a context in which negative and positive liberties are balanced, regulatory policies have double meaning. Thus, there should be a balance between the establishment of necessary rules for social protection and limits for them not to violate individuals’ rights.

## INTRODUCTION

Public health strategies that aim at increasing population’s health levels have received great attention in recent years. A variety of intersectoral initiatives have been implemented, embracing, among others, public spaces, urban mobility, water, food and work^1,2^. Regulatory policies to facilitate the access to food supplementation, safe water and immunization, as well as to improve traffic safety and tobacco control, have been considered of inestimable value in increasing life expectancy worldwide^3^. Regarding oral health, the public policy on the fluoridation of public water supply has been the most important achievement in the USA^4^,Brazil^5^ and Australia^6^.

These initiatives entail a complex political process in which actors from different sectors must act in a health-related problem, demanding the articulation of a set of shared strategies and activities. Among other aspects, the arrangement of these sectors faces important barriers due to different political principles and moral values that guide the actors^7^.Regarding water fluoridation, this conflicting scenario seems to reflect the dispute between two conceptions of liberty^8^.

This study aims to discuss these conceptions and postulate their interdependent and complementary relationship. To do so, we will use positive and negative concepts of liberty, distinction that gained prominence in the 1950s Isaiah Berlin’s work^9^, whose influence on the political theory of liberty remains until the beginning of the 21st century. In the last section, we try to show how the regulatory policy on fluoridation is articulated with the notion of interdependence and complementarity between the two dimensions of liberty.

### THE NEGATIVE CONCEPT OF LIBERTY

This idea concerns the area within which the subject (a person or group of people) is, or should be, left to do or be whatever they want to do or be, without other people’s interference. If this area is incurred by a third party, the subject or group may be under coercion. If coercion degree increases indefinitely, subject becomes subjected, enslaved, a being deprived of liberty. Thus, the larger the non-interference area, the greater the liberty. However, this area shall not be unrestricted as it would imply a scenario in which all men could interfere without constrains in other men. This “natural” liberty would lead to social chaos, and men’s basic needs would not be satisfied; or else, the liberty of the weak would be suppressed by the strong. Liberty, in this sense, means liberty “from,” e.g. the absence of interference beyond a dynamic, but always recognizable, boundary^9^.

Such concept is commonly associated with constitutional liberties typical of liberal-democratic societies, such as freedom of movement, freedom of religion and freedom of expression. Therefore, it is related to civil rights and classical liberalism, that is: the notion that the State should not interfere in citizens liberty and property. Furthermore, their interests should be considered legitimate as long as they do not threaten other citizens’ rights^10^. It is the most widespread concept within our professional environment.

### THE POSITIVE CONCEPT OF LIBERTY

This notion refers to the source of control or interference that persuade someone to do or be one thing instead of another. It concerns less in *which* area the subject is free to do or be, and more to those *who* define what the subject or group is or is not, what they should or should not do. That is, by whom the subject or group is governed. Therefore, the more the subject owns himself and is independent of external forces, the greater his liberty, autonomy and ability to self-determine. Liberty, in this sense, means liberty “to” determine your own fate or those of others. It implies self-realization or self-determination, whether individual or a collective^9^.

Positive liberty is often seen as achieved necessarily through a collectivity: a tribe, an identity group, a church, a party, a State, or other entity identified as being the “true” self. This collectivity transfers its common and unique will to its members, more and less avid for their aspirations. Through participation the community would exercise collective control over its own matters, according to the “general will” of those belonging to it. This entity would actively seek the necessary conditions for individuals to become self-reliant or achieve self-realization.

However, the pursuit of these conditions could not be unlimited, as it could turn into a brutal tyranny. The mechanism which would avoid the concentration of power and oppression is: a democratic constitution and a set of safeguards against the government arbitrary power, including the separation of powers, weights, counterweights and the exercise of civic virtues by citizens. The social welfare and the idea of a universal basic income are supported on these assumptions. The positive concept of liberty is usually associated with political and social rights, freedom to participate in the government and deliberate and supervise the allocation of public resources^10^.

## FINAL CONSIDERATIONS

The unequal distribution of the causes, risks and damages to health is killing people on a large scale. Far from a “natural” phenomenon, it results from a toxic combination of precarious social programs and policies, unfair economic arrangements and uneven power distribution^11^. Although the distinction between the positive and negative concepts is clear, there are still overlapped zones that motivate an extensive debate among liberty theorists. Berlin^9^argues that considering liberty a value in itself does not turn it into the most important and unique purpose of a human being. For him, there are values as or even more important, such as equality and justice, which provide the basis for the prevailing ethical-political orientations and distinguish a civilized man from a barbarian. Both negative and positive concepts of liberty can validly defend a social legislation, a well-being society and socialism. If it was not done so often historically, it was because the evil against which the negative concept of liberty was usually directed to was not in relation to an uncontrolled market economy, but rather to the political despotism, and both forms of despotism are related to social injustice^12^.

These different orientations provoke the diversity of meanings on the positive and negative concepts of liberty present in the academic world^13^and the political arena. Whereas for some political actors these concepts deny each other, for others they are complementary.

Among those who believe that both concepts rival each other, there are actors who defend individual interest precedence over general interest; and actors who support the relevance of the general interest over individual. Those who place concepts in opposing poles consider individual rights more important than political and social rights, as if the liberal citizen protecting his own interests and the republican citizen protecting the general interests do not represent two interdependent dimensions of the same statute. The most extreme conception does not recognize that individual rights are only guaranteed within a society in which collective action is effective in the creation of liberal and democratic institutions that ensure the application of both individual and collective rights comprising each citizen’s right.

Within this bipolar view, intersectoral actions are falsely portrayed as a choice between individual responsibility and liberty restriction. Such perspective is commonly found on the internet and social networks. The expansion of this view has led to the multiplication of thousands of electronic pages and messages seen as trustworthy information sources by an increasing number of users. In this framework, there is no room for reconciliation between the general and individual interest. The consequences on population’s health, resulting from the omission of health authorities, are not seen as a regulatory option. As if the absence of a cigarette-free workplace was not considered a political decision responsible for greater exposure of workers to carcinogens and increased risk of acute myocardial disease^14^.

Regarding the debate on the appropriate role of regulatory approaches to improve health, overcoming this bipolar view and recognizing both the rights that protect citizens against a powerful State and the rights that protect the State against powerful citizens are crucial tasks for health leaders. The Universal Declaration of Human Rights, by embracing both dimensions, is an important instrument for guiding these decisions.

Some researchers describe the long history of how water fluoridation has become a public health strategy and a regulatory activity in the United States.^8^ Disputes, from an ethical-political point of view, and conflicts, from the scientific point of view and the interpretation of advances in epidemiological and sanitary knowledge, commonly reflected personal battles arising from unfair accusations and leading to ruined reputations. In a country which over 70% of the population benefits from the policy of fluoridation water, lawsuits in the U.S. courts appealing for the interruption of the measure have been denied for various reasons. Among them, there is the justification that the statutes for fluoridation implementation are state-regulation valid exercises. Private rights are not absolute and must be modulated according to collective objectives acquired legitimately and regarding the means of law enforcement^15^.

If rights complemented each other, fluoridation would not violate individual rights of choice for at least two reasons. First, because safe water is rich in mineral salts. Fluoride is one of those elements, naturally contained in surface and underground springs. Thus, it is not a laboratory manufactured element which is unusual to water nature. Second, because water, once captured and treated, has its content adjusted for a concentration considered optimal for caries prevention, at levels below the maximum value allowed for its potability. That is, the adjustment value is about two times lower than the value up from which water would be considered unfit for human health.

Consequently, if there was no adjustment of fluoride concentration in public water supply, the risk of caries lesions would probably increase, especially among the most numerous and of worse socioeconomic conditions social segments. Values regarding individual freedom, collective rights, equal opportunities and social justice enable the adoption, by democratic means, of norms in favor of these social segments. Because it is a safe, cost-effective and comprehensive measure of public health, its implementation can be considered a manifestation of a free and informed decision under the two concepts of liberty.

On the one hand, it expresses the individual free decision resulting from the acknowledgement that water, as electricity and the breathing air, is a public rather than a private good. Thus, if a citizen acquires this good, other citizens are not unable to obtain it too. A subject cannot go to a store and choose the X or Y water network, as if it was a piece of clothing or a car. Because it is a public good, providing water for new homes and consumers has practically null additional costs. Individuals are hardly ever excluded from its consumption, and some may even be exempted from paying for it. Such externalities are considered positive by being much greater than an individual benefit. On the other hand, it expresses a collective free decision related to the acknowledgement that safe water is that which physicochemical and microbiological characteristics are known and controlled. As a factor of protection against diseases, ensuring safe water is of both general and individual interest and, therefore, comprises the exercise of the right to health as a universal human right.

In a context in which both concepts of liberty are balanced, regulatory policies have double meaning. While mentoring the establishment of what can be done and how, they learn how to adapt to the progress of evidences and the needs arising from contemporary changes. Their wisdom lies in finding a balance between establishing the necessary rules for public protection and boundaries for these rules not to violate individuals’ rights^16^.

This study addressed particularly water fluoridation. However, as the refusal to take preventive measures, such as childhood immunizations, was associated with the refusal to use fluoride in dental caries prevention, the study might be pertinent for the reflection of other public policies^17^.

## References

[1] 1. World Health Organization. Intersectoral Action on Health: a path for policy-makers to implement effective and sustainable action on health: discussion paper. In: First Global Ministerial Conference on Healthy Lifestyles and Noncommunicable Disease Control; 2011 April 28-29; Moscow, Russia. WHO Centre for Health Development; 2011 [cited 2018 Oct 20]. Available from: https://www.who.int/nmh/publications/ncds_policy_makers_to_implement_intersectoral_action.pdf

[2] 2. Freiler A, Muntaner C, Shankardass K, Mah CL, Molnar A, Rehahy E, et al. Glossary for the implementation of Health in All Policies (HiAP). J Epidemiol Community Health. 2013;67(12):1068-72. 10.1136/jech-2013-202731 PMC3841750 23986493

[3] 3. Centers for Disease Control and Prevention. Ten Great Public Health achievements --- Worldwide, 2001-2010. MMWR Morb Mortal Wkly Rep. 2011;60(24);814-8. 21697806

[4] 4. Centers for Disease Control and Prevention. Ten Great Public Health achievements -- United States, 1900-1999. MMWR Morb Mortal Wkly Rep. 1999;48(12);241-3. 10220250

[5] 5. Ramires I, Buzalaf MAR. A fluoretação da água de abastecimento público e seus benefícios no controle da cárie dentária: cinqüenta anos no Brasil. Cienc Saude Coletiva. 2007;12(4):1057-65. 10.1590/S1413-81232007000400027 17680164

[6] 6. Public Health Association of Australia. Top 10 public health successes over the last 20 years, Canberra: PHAA; 2018 [cited 2018 Oct 20]. (PHAA Monograph Series, 2). Available from: https://www.phaa.net.au/documents/item/3241

[7] 7. Howlett M, Ramesh M, Perl A. Política pública: seus ciclos e subsistemas: abordagem integral. Rio de Janeiro: Elsevier; 2013. O contexto da política pública; p. 57-71.

[8] 8. Freeze RA, Lehr JH. The fluoride wars: how a modest public health measure became America’s longest-running political melodrama. Hoboken, NJ: John Wileyand Sons; 2009.

[9] 9. Berlin I. Two concepts of liberty. In: Berlin I, Hardy H, Harris I. Liberty: incorporating four essays on liberty. Oxford: Oxford University Press; 2009. p.155-65.

[10] 10. Bresser-Pereira LC. Citizenship and Res Publica: the emergence of republican rights. Citizenship Stud. 2002;6(2):145-64. 10.1080/13621020220142941

[11] 11. World Health Organization, Commission on Social Determinants of Health. Closing the gap in a generation: health equity through action on the social determinants of health: final report. Geneva: WHO; 2008 [cited 2018 Oct 20]. Available from: https://www.who.int/social_determinants/thecommission/finalreport/en/

[12] 12. García Amado JA. El liberalismo de Isaiah Berlin: la libertad, sus formas y sus límites. Derechos Libertades. 2006;(14):41-88.

[13] 13. Elford G. Reclaiming two concepts of liberty. Polit Philos Econ. 2012;12(3):228-46. 10.1177/1470594X12460643

[14] 14. Chokshi DA, Stine NW. Reconsidering the politics of Public Health. JAMA. 2013;310(10):1025-6. 10.1001/jama.2013.110872 23975269

[15] 15. Mendonza RL. Fluoride-treated water and the merit goods. Water Policy. 2011;13(1):38-52. 10.2166/wp.2010.127

[16] 16. Roemer R. Water fluoridation: public health responsibility and the democratic process. Am J Public Health Nations Health. 1965;55(9):1337-48. 10.2105/ajph.55.9.1337 PMC1256473 14334756

[17] 17. Chi DL. Caregivers who refuse preventive care for their children: the relationship between immunization and topical fluoride refusal. Am J Public Health. 2014;104(7):1327-33. 10.2105/AJPH.2014.301927 PMC4056200 24832428

