# Supplementary material for: Liberties, rights, public policies and water fluoridation
Source: Rev Saude Publica. 2020 May 22;54:51. doi: 10.11606/s1518-8787.2020054001804 (PMC7244233; doi:10.11606/s1518-8787.2020054001804)
Supplement: Translation

# Translation: Liberdades, direitos, políticas públicas e a fluoretação da água

**Authors:** Antonio Carlos de Souza, Paulo Frazão

**Affiliations:** I Brasil Universidade de São Paulo. Faculdade de Saúde Pública. Programa de Pós-Graduação em Saúde Pública. São Paulo, SP, Brasil; II Brasil Universidade de São Paulo. Faculdade de Saúde Pública. Departamento de Política, Gestão e Saúde. São Paulo, SP, Brasil

**Keywords:** Políticas Públicas de Saúde, Direito Administrativo, Direito Sanitário, Direitos Socioeconômicos, Liberdade

## Abstract

**OBJETIVO:**

Discutir os conceitos negativo e positivo de liberdade e postular sua relação de interdependência e complementariedade na avaliação da política pública intersetorial, tomando como caso a fluoretação da água.

**MÉTODO:**

Descrevem-se os alcances e limites relativos à distinção formulada por Isaiah Berlin nos anos 1950, demonstrando sua validade para enfrentar os efeitos nocivos decorrentes tanto de uma economia de mercado sem controle quanto de um regime político autocrático.

**RESULTADOS:**

Reconhece-se que são igualmente cruciais tanto os direitos que protegem os cidadãos contra um Estado poderoso quanto os direitos que protegem o Estado contra os cidadãos poderosos.

**CONCLUSÃO:**

Argumenta-se que, em um contexto de equilíbrio entre a liberdade negativa e positiva, a política regulatória tem duplo sentido, e deve buscar um balanço entre o estabelecimento de regras necessárias para a proteção do público e de limites além dos quais as regras não devem violar os direitos dos indivíduos.

## INTRODUÇÃO

Estratégias de saúde pública voltadas à elevação dos níveis de saúde das populações têm recebido grande atenção nos últimos anos, e uma variedade de iniciativas de políticas públicas intersetoriais têm sido implementadas, abrangendo, entre outros temas, espaços públicos, mobilidade urbana, água, alimentação e trabalho^1,2^. Políticas regulatórias para facilitar o acesso à suplementação alimentar, água segura e imunização, como também para melhorar a segurança no trânsito e o controle do tabaco, têm sido reconhecidas como de valor inestimável no aumento da expectativa de vida no mundo^3^. Do ponto de vista da saúde bucal da população, a política pública da fluoretação da água de abastecimento público tem sido a conquista mais importante nos EUA^4^, no Brasil^5^ e na Austrália^6^.

Essas iniciativas envolvem um processo político complexo, que inclui atores pertencentes a diferentes setores em torno de um problema relacionado à saúde que exige a articulação de um conjunto de estratégias e atividades compartilhadas. Tal arranjo entre os setores enfrenta importantes barreiras relacionadas, entre outros aspectos, aos diferentes princípios políticos e valores morais que orientam os atores^7^. No caso da fluoretação da água, esse contexto conflitivo parece refletir a disputa entre duas concepções de liberdade^8^.

O objetivo deste comentário é discutir essas concepções e postular sua relação de interdependência e complementariedade. Para isso utilizaremos os conceitos positivo e negativo de liberdade, uma distinção que ganhou proeminência na obra de Isaiah Berlin nos anos 1950^9^, cuja influência sobre a teoria política da liberdade se mantém no início do século XXI. Na última seção, procuramos demonstrar como a política regulatória da fluoretação se articula à noção de interdependência e complementariedade entre as duas dimensões de liberdade.

## O CONCEITO NEGATIVO DE LIBERDADE

Essa ideia diz respeito à área dentro da qual o sujeito (uma pessoa ou grupo de pessoas) é, ou deve ser, deixado para fazer ou ser o que ele quiser fazer ou ser, sem a interferência de outras pessoas. Se essa área é contraída por terceiros, o sujeito ou grupo pode estar sendo coagido. Se o grau da coação aumenta indefinidamente, o sujeito se transforma em sujeitado, escravizado, um sem-liberdade. Portanto, quanto maior a área de não interferência, maior seria a liberdade. Entretanto, é reconhecido que essa área não pode ser irrestrita, pois implicaria uma situação na qual todos os homens poderiam interferir sem limites nos outros homens, e esse tipo de liberdade “natural” levaria ao caos social, no qual as necessidades mínimas dos homens não seriam satisfeitas; ou então as liberdades dos fracos seriam suprimidas pelos fortes. Liberdade, nesse sentido, significa liberdade “de”: ausência de interferência além de uma fronteira dinâmica, mas sempre reconhecível^9^.

Essa ideia é comumente associada às liberdades constitucionais típicas de sociedades liberal-democráticas, tais como liberdade de movimento, liberdade de religião e liberdade de expressão. Está, portanto, conectada aos direitos civis e ao liberalismo clássico, ou seja, à noção de que o Estado não deve interferir na liberdade e propriedade dos cidadãos, cujos interesses devem ser considerados legítimos desde que não ameacem os direitos de outros cidadãos^10^, sendo a acepção mais difundida em nosso meio profissional.

## O CONCEITO POSITIVO DE LIBERDADE

Essa noção refere-se à fonte de controle ou interferência que pode determinar alguém a fazer, ou ser, uma coisa em vez de outra. Diz respeito menos à área em que o sujeito é livre para fazer ou ser, e mais a quem define o que o sujeito ou grupo é ou não é, o que deve ou não fazer, por quem o sujeito ou grupo é governado. Portanto, quanto mais o sujeito é dono de si mesmo, e independente de forças externas, maior sua liberdade, sua autonomia e sua capacidade de se autodeterminar. Liberdade, nesse sentido, significa liberdade “para” determinar o próprio destino ou o destino de outros. Implica autorealização ou autodeterminação, seja de um indivíduo ou de uma coletividade^9^.

A liberdade positiva tem sido frequentemente vista como necessariamente alcançada por meio de uma coletividade, seja uma tribo, um grupo identitário, uma igreja, um partido, um Estado ou outra entidade identificada como sendo o “verdadeiro” eu, que transfere sua vontade coletiva e única aos seus membros mais e menos ávidos por suas aspirações. Seria por meio da participação que a comunidade exerceria controle coletivo sobre seus próprios assuntos de acordo com a “vontade geral” daqueles que com ela estabelecem laços de pertencimento. Esse ente buscaria ativamente as condições necessárias para que os indivíduos fossem autossuficientes ou conseguissem alcançar a autorrealização.

Adverte-se, entretanto, que a busca dessas condições não poderia ser ilimitada, sob pena de se transformar numa tirania brutal. Uma constituição democrática e uma série de salvaguardas contra o poder arbitrário de um governo, incluindo a separação de poderes, pesos, contrapesos e o exercício de virtudes cívicas por parte dos cidadãos, seria o mecanismo pelo qual poderia ser evitada a concentração de poder e a opressão. O Estado de bem-estar social tem sido defendido nessa base, assim como a ideia de uma renda básica universal. O conceito positivo de liberdade geralmente é associado aos direitos políticos e sociais, à liberdade de participar do governo, deliberar e fiscalizar a alocação do recurso público^10^.

## CONSIDERAÇÕES FINAIS

A distribuição desigual das causas, dos riscos e dos danos à saúde está matando pessoas em grande escala, e longe de um fenômeno “natural”, é resultado de uma combinação tóxica de programas e políticas sociais precárias, arranjos econômicos injustos e poder desigualmente distribuído^11^. Embora a distinção entre os conceitos positivo e negativo seja clara, existem espaços de sobreposição que têm motivado um extenso debate entre os teóricos da liberdade. Berlin^9^sustenta que a premissa de considerar a liberdade como um valor em si mesma não a torna a mais importante e única finalidade do ser humano. Para ele, existem valores tão ou mais importantes, como a igualdade e a justiça, que fornecem as bases das diferentes orientações ético-políticas vigentes em uma sociedade e que distinguem um homem civilizado de um bárbaro. A defesa de uma legislação social, de uma sociedade do bem-estar e do socialismo, pode ser feita com validade, tanto a partir da noção negativa quanto a partir da noção positiva de liberdade. Se historicamente isso não foi feito com tanta frequência, foi porque o tipo de mal contra o qual a arma do conceito de liberdade negativa foi usualmente dirigida não foi em relação a uma economia de mercado sem controle, mas em relação ao despotismo político, e ambas formas de despotismo estão associadas à injustiça social^12^.

Essas distintas orientações dão origem à diversidade de acepções atribuídas aos conceitos positivo e negativo de liberdade presentes no mundo acadêmico^13^e na arena política. Para alguns atores políticos, essas concepções se rivalizam, enquanto para outros, se complementam.

Entre aqueles que as rivalizam, pode-se identificar atores que defendem a precedência do interesse individual sobre o interesse geral e atores que sustentam a relevância do interesse geral sobre o interesse individual. Aqueles que as posicionam em polos opostos consideram os direitos individuais mais importantes do que os direitos políticos e sociais, como se o cidadão liberal que protege seus próprios interesses e o cidadão republicano que protege os interesses gerais não representassem duas dimensões interdependentes do mesmo estatuto. Na concepção mais extrema, não se reconhece que os direitos individuais só podem ser garantidos dentro de uma sociedade em que a ação coletiva é eficaz na criação de instituições liberais e democráticas que assegurem tanto sua aplicação quanto os direitos coletivos que também são direitos de cada cidadão individual.

Nessa visão bipolar, as políticas intersetoriais de saúde são falsamente retratadas como uma escolha entre a responsabilidade individual versus a restrição de liberdade. Essa perspectiva é facilmente encontrada na internet e nas redes sociais, cuja expansão propiciou a multiplicação de milhares de páginas eletrônicas e mensagens vistas como fontes de informação dignas de confiança por um número crescente de usuários. Nessa visão, não há espaço para a conciliação entre o interesse geral e o interesse individual. As consequências para a saúde da população decorrentes da omissão das autoridades sanitárias não são vistas como uma opção regulatória. É como se a ausência de um local de trabalho livre de cigarro não fosse considerada uma decisão política que engendra maior exposição do trabalhador a agentes cancerígenos e risco aumentado de doença miocárdica aguda^14^.

Superar essa visão bipolar e reconhecer que são igualmente necessários tanto os direitos que protegem os cidadãos contra um Estado poderoso quanto os direitos que protegem o Estado contra os cidadãos poderosos é, portanto, uma tarefa crucial para as lideranças sanitárias, no importante e legítimo debate sobre o papel apropriado das abordagens regulatórias para melhorar a saúde das populações. Por abranger ambas as dimensões, a Declaração Universal dos Direitos Humanos constitui-se num importante instrumento para orientar tais decisões.

Alguns pesquisadores detalham cenas de uma longa história de como a fluoretação da água se tornou uma estratégia de saúde pública e uma atividade regulatória nos Estados Unidos^8^. As disputas do ponto de vista ético-político, e os conflitos do ponto de vista científico e da interpretação dos avanços do conhecimento epidemiológico e sanitário, em muitos casos refletiam batalhas de personalidade, tendo origem em acusações injustas e terminando com reputações arruinadas. Nesse país onde mais de 70% da população é beneficiada pela política da fluoretação da água de abastecimento público, processos judiciais nas cortes americanas de apelação para a interrupção da medida têm sido negados por várias razões, entre as quais a justificativa de que os estatutos para a instituição da fluoretação são exercícios válidos de regulação estatal. Direitos privativos não são absolutos e devem ser modulados em nome de objetivos coletivos legitimamente adquiridos e relacionados aos meios de aplicação da lei^15^.

Numa visão na qual os direitos se complementam, a fluoretação não violaria direitos individuais de escolha, por pelo menos dois motivos. Primeiro, porque água segura para a saúde é água rica em sais minerais, e o fluoreto é um desses elementos que estão naturalmente presentes nos mananciais superficiais e subterrâneos. Portanto, não é um elemento fabricado em laboratório estranho à natureza da água. Segundo, porque a água, ao ser captada e tratada, tem seu teor ajustado para a concentração considerada ótima na prevenção da cárie dentária em níveis inferiores ao valor máximo permitido do ponto de vista da sua potabilidade, ou seja, o valor de ajuste é cerca de duas vezes mais baixo que o valor acima do qual a água seria considerada imprópria para a saúde humana.

Assim, não promover o ajuste da concentração do fluoreto na água de abastecimento público pode significar aumento do risco de novas lesões de cárie dentária, principalmente entre os segmentos sociais mais numerosos e de piores condições socioeconômicas. Valores concernentes à liberdade individual, ao direito coletivo, à igualdade de oportunidades e à justiça social permitem sustentar a aprovação, por meios democráticos, de normas em favor dos segmentos sociais mais numerosos, excluídos e de piores condições socioeconômicas. Por se constituir em uma medida de saúde pública segura, barata, custo-efetiva e abrangente, sua implementação pode ser considerada manifestação de uma decisão livre e informada sob as duas perspectivas de liberdade mencionadas.

De um lado, ela expressa a decisão livre do indivíduo resultante da compreensão de que a água, como a eletricidade e o ar que respiramos, é um bem público e não um bem do tipo privado, em que a aquisição por um cidadão implica na impossibilidade desse bem ser obtido por outro cidadão; o sujeito não pode ir até uma loja e escolher a rede de água X ou Y, como faz com uma peça de roupa ou um automóvel. Por ser um bem público, o custo adicional de provimento da água para novas moradias e consumidores é praticamente zero. Dificilmente algum indivíduo é excluído do seu consumo, e alguns usuários podem inclusive ser dispensados de pagar por esse bem. Essas externalidades são consideradas positivas porque são muito maiores que um benefício individual. Por outro lado, expressa uma decisão livre coletiva associada à compreensão de que água segura é água cujas características físico-químicas e microbiológicas são conhecidas e controladas. Por ser fator de proteção contra as doenças, assegurar água segura é tanto de interesse geral quanto de interesse individual e, portanto, exercício do direito à saúde enquanto um direito humano universal.

Em um contexto de equilíbrio entre ambas acepções de liberdade, a política regulatória tem duplo sentido. Ao mesmo tempo em que ela é mentora na definição do que pode ser feito e como, ela é aprendiz em sua prontidão para se adaptar, conforme o avanço das evidências e as necessidades decorrentes das mudanças contemporâneas. Sua sabedoria está em encontrar um balanço entre o estabelecimento de regras necessárias para a proteção do público e de limites além dos quais as regras não devem violar os direitos dos indivíduos^16^.

Este comentário abordou especificamente a fluoretação da água, mas como a recusa do cuidador diante de medidas preventivas como a oferta de imunizações infantis foi associada à recusa ao uso de fluoreto para prevenção da cárie dentária, admite-se que possa ser útil para a reflexão de outras políticas públicas^17^.
